# Characteristics of COVID‐19 delirium intervened by a psychiatric liaison team in the first 2 years of the COVID‐19 pandemic in Japan

**DOI:** 10.1111/pcn.13456

**Published:** 2022-08-28

**Authors:** Kyohei Otani, Atsumi Miura, Hiroyuki Miyai, Haruko Fukushima, Kunitaka Matsuishi

**Affiliations:** ^1^ Department of Psychiatry Kobe City Medical Center General Hospital Kobe Japan

Delirium is common in COVID‐19 patients, and such patients have characteristics such as severe pneumonia with inflammatory reactions; prolonged intubation, respiratory management, and sedation; disuse syndrome; central nervous system effects; behavioral restrictions owing to droplet infection and airborne infection in poorly evacuated areas.^1^

In the first 2 years of the pandemic, 1336 patients with COVID‐19 were admitted in Kobe City Medical Center General Hospital, which provides emergency care for all patients from primary to tertiary emergencies 24 h a day, 365 days a year without refusal. During the same period, 1336 patients were referred to the liaison team, including 149 COVID‐19‐related referrals. Of these, 100 were referred for delirium (COVID‐19 pneumonia with delirium [COV‐pd] group).

Table 1 shows the characteristics of the COV‐pd group, which included four subtypes – 41, 32, 13, and 14 patients with the wild, alpha, delta, and omicron strains, respectively, and their comparison with 63 patients who comprised the non‐COVID‐19 pneumonia with delirium (non‐COV‐pd) group, which included 21 patients with aspiration pneumonia, 19 patients with bacterial pneumonia, 16 patients with interstitial pneumonia, and seven with others. The liaison team intervened during the same period.

**Table 1:** Comparison of the characteristics of COVID‐19 pneumonia patients with delirium (COV‐pd) and non‐COVID‐19 pneumonia patients with delirium (non‐COV‐pd) who were intervened by the liaison team in Kobe City Medical Center General Hospital

	COV‐pd (*N* = 100)	Non‐COV‐pd (*N* = 63)	*P*‐value
Subtypes, *n* (%)	41 (41.0%) W	21(33.3%) AsP	–
	32 (32.0%) α	19(30.2%) BP	
	13 (13.0%) δ	16(25.4%) IP	
	14 (14.0%) ο	7(11.1%) others#	
Age, mean (SD)[years]†	70.8 (11.8)	82.0 (6.4)	<0.001**
Age range [years]	38–97	66–98	‐
Male sex, *n* (%)‡	68 (68%)	43 (68.3%)	1.00
Endotracheal intubation, *n* (%)§	57 (57.0%)	11 (17.5%)	<0.001**
ICU admission, *n* (%)§	61 (61.0%)	19 (30.2%)	<0.001**
Transvenous sedative use, *n* (%)§	55 (55.0%)	11 (17.5%)	<0.001**
Use of nasal high‐flow, *n* (%)§	24 (24.0%)	16 (25.4%)	0.85
Behavioral restrictions, *n* (%)§	99 (99.0%)	48 (76.2%)	<0.001**
Physical restraints, *n* (%)§	90 (90.0%)	48 (76.2%)	0.025*
Comorbidities of chronic diseases, *n* (%)§	92 (92.0%)	63 (100%)	0.024*
High blood pressure, *n* (%)§	57 (57.0%)	28 (44.4%)	0.14
Type 2 diabetes mellitus, *n* (%)§	27 (27.0%)	15 (23.8%)	0.71
Dyslipidemia, *n* (%)§	16 (16.0%)	9 (14.3%)	0.82
Chronic renal failure, *n* (%)§	14 (14.0%)	3 (4.8%)	0.06
Atrial fibrillation, *n* (%)§	7 (7.0%)	10 (15.9%)	0.11
Bronchial asthma, *n* (%)§	5 (5.0%)	4 (6.3%)	0.73
Chronic obstructive pulmonary disease, *n* (%)§	4 (4.0%)	8 (12.7%)	0.06
Previous cerebral infarction, *n* (%)§	4 (4.0%)	8 (12.7%)	0.06
Alcoholic liver disease, *n* (%)§	4 (4.0%)	4 (6.3%)	0.71
Charlson Comorbidity Index, (SD, points) (%)†	0.79 (1.16)	1.83 (2.21)	0.001**
Charlson Comorbidity Index range (points)	0–5	0–9	–
Encephalopathy, *n* (%)§	10 (10.0%)	0 (0.0%)	0.007**
Antipsychotic drug use, *n* (%)§	95 (95.0%)	55 (87.3%)	0.13
Oral benzodiazepines use, *n* (%)§	16 (16.0%)	2 (3.2%)	0.010*
Mortality, *n* (%)§	18 (18.0%)	14 (22.2%)	0.24

* Statistically significant difference (*P* < 0.05).

** Statistically significant difference (*P* < 0.01).

^†^ Analyzed by the *t* test.

^‡^ Analyzed by the χ^2^‐test.

^§^ Analyzed by the Fisher's exact probability test.

COV‐pd: Hundred patients comprised the COVID‐19 pneumonia with delirium group and were treated by the liaison team from 3 March 2020, to 14 March 2022, which was the period between the first to sixth pandemic waves.

Non‐COV‐pd: Sixty‐three patients comprised the non‐COVID‐19 pneumonia with delirium group which was intervened during the same period.

Comorbidities are noted in order of frequency as investigated in the medical records database. The Charlson Comorbidity Index was calculated using records from the Japanese Diagnosis Procedure Combination (DPC) data.

SD, standard deviation; W, wild type strain; α, alpha type strain; δ, delta type strain; ο, omicron type strain; AsP, aspiration pneumonia; BP, bacterial pneumonia; IP, interstitial pneumonia; #, empyema pneumonia, organizing pneumonia, and drug‐induced pneumonia.

There was no significant difference in mortality between the COV‐pd and non‐COV‐pd groups. This result is surprising since from the significant differences in the rates of endotracheal intubation, ICU admission, and transvenous sedative use, it can be inferred that pneumonia was more severe in the COV‐pd group.

The COV‐pd group also had a lower mean age and significantly higher rates of behavioral restrictions, including the use of physical restraints, and oral benzodiazepines, such as lorazepam. The non‐COV‐pd group had a 100% rate of comorbidities (Table 1) and significantly higher score on the Carlson Comorbidity Index (CCI).^2^ To compare the severity of the comorbidities between the two groups, the CCI was calculated using records from the Japanese Diagnosis Procedure Combination data.^3^

Patients in the COV‐pd group were younger, had critical complications, including COVID‐19 encephalopathy, more severe pneumonia, and a higher percentage of them were behaviorally restricted; whereas, the non‐COV‐pd patients were older, had more chronic diseases, and they had a higher score on the CCI. COVID‐19 pneumonia is more likely to cause delirium due to the severity of the pneumonia itself, COVID‐19 encephalopathy, and behavioral restrictions. Therefore, successful acute treatment of COVID‐19 pneumonia will improve pneumonia itself and complications and reduce behavioral restrictions, resulting in improvement of delirium. However, this is limited by the fact that pneumonia patients without delirium have not been studied. Also, our hospital accepts all patients without refusal, which may have led to the admission of more patients with COVID‐19 severe pneumonia and delirium. As a single‐center study, our results are limited by the fact that the severity of delirium varies with the severity of patients admitted to the hospital.

The liaison team intervened in 10 patients (nine males and one female; mean age, 66.3 ± 11.4 years [range, 40–83 years]) with delirium associated with COVID‐19 encephalopathy; their delirium was overlooked in the early stages of treatment because the symptoms of pneumonia required immediate intervention. The frequency of COVID‐19 encephalopathy varies from 36.4 to 82.3% and is responsible for delirium, disturbed consciousness, epileptic seizures, and syncope.^4^, ^5^

Patients in the COV‐pd group received more behavioral restrictions, either quarantine to the isolation ward or physical restraints, or both, than patients in the non‐COV‐pd group. Physical restraints are usually used to prevent medical accidents, such as falls and stumbles, and to protect patient safety. Physical restraints may also have to be used in cases of COVID‐19 delirium to prevent the spread of infection, which has been reported to increase in hospitals that have COVID‐19 patients,^6^ but they exacerbate delirium.

Mortality was not significantly different between the two groups; however, the COV‐pd group showed more severe pneumonia and a higher complication rate of encephalopathy in addition to more incidences of physical restraint use and other behavioral restrictions and use of benzodiazepines. Our hospital did not have a delirium management protocol specifically for COVID‐19 related delirium. The use of (non‐) benzodiazepines is prohibited for patients at a high risk of delirium because they trigger it; however, several guidelines published in other countries^7^, ^8^ recommend the administration of benzodiazepines when antipsychotics are ineffective, which may indicate the difficulty in dealing with COVID‐19 delirium. Although opioids and benzodiazepines have been reported to worsen delirium, these drugs are effective in relieving symptoms such as respiratory distress and treating hypoactive delirium at the end of life, particularly in patients with cancer.^9^

## Disclosure statement

The authors declare no conflicts of interests.

## Ethics Approval Statement

This study was conducted in accordance with the principles of the Declaration of Helsinki and was approved by the Institutional Review Board of the Kobe City Medical Center General Hospital.

## Consent to Participate

An opt‐out method was used to obtain consent.
